# Sleep patterns and cardiometabolic risk in schoolchildren from Cuenca, Spain

**DOI:** 10.1371/journal.pone.0191637

**Published:** 2018-01-23

**Authors:** Lidia Lucas-de la Cruz, Noelia Martín-Espinosa, Iván Cavero-Redondo, Alberto González-García, Ana Díez-Fernández, Vicente Martínez-Vizcaíno, Blanca Notario-Pacheco

**Affiliations:** 1 Universidad de Castilla-La Mancha, Faculty of Nursing, Cuenca, Spain; 2 Universidad de Castilla-La Mancha, Health and Social Research Center, Cuenca, Spain; 3 Universidad de Castilla-La Mancha, Faculty of Nursing, Toledo, Spain; 4 Facultad de Ciencias de la Salud, Universidad Autónoma de Chile, Talca, Chile; Universidad de las Palmas de Gran Canaria, SPAIN

## Abstract

Sleep seems to have a significant influence on the metabolic syndrome (MetS). However, results in this association are still inconsistent in children. The aim of this study was to examine the influence of sleep characteristics in the MetS (index and factors) in Spanish children. Cross-sectional study including a sample of 210 children aged 8-to-11-years belonging to 20 schools from the province of Cuenca, Spain was conducted. Cardiometabolic risk and actigraphy sleep patterns were determined and analysed using correlation coefficients, ANCOVA models and a propensity score derivation model. Overall, children in the lower time in bed category and those who went to bed later (> 23:15h) showed worse values in the cardiometabolic profile and risk index. Differences were observed when the total time in bed was below 9h 15mins. Our study shows that short sleep duration could be a risk factor for cardiometabolic risk in children, and bedtime may independently influence this risk. In addition, our data suggests that children’s sleep hygiene should be incorporated in parenting educational programs.

## Introduction

Metabolic syndrome (MetS) has been conceptualized as a cluster of cardiometabolic risk factors (abdominal obesity, insulin resistance, dyslipemia and elevated blood pressure). [1] Epidemiologic studies have consistently shown that physically active children have a better cardiometabolic profile than those who are less active [2–4] and because of the increase of obesity in children, the interest in MetS in this population is growing.

The association between sleep duration and several adverse health outcomes including cardiovascular diseases has been repeatedly reported. [5–8] Particularly, a short sleep duration has been associated with decreased insulin sensitivity, lipid profile abnormalities (increased weight or adiposity) and increasing blood pressure (BP) in children and adolescents. [9–13]

Recent research has addressed the impact of sleep timing (combination of bedtime and wake-up time) on health related quality of life. [14] Bedtime has a greater impact on children’s sleep duration than wake-up time does, [15–16] it is more modifiable than wake-up time, and thus, it is likely the behavioural target of clinicians and parents. Several studies have analysed the effect of bedtime on the risk of obesity in schoolchildren; [17] for example, evidence shows that a later bedtime in children is associated with a higher BMI and a risk for overweight/obesity. [15, 18, 19]

When analysing observational or quasi-experimental studies, propensity score matching is a useful strategy to reduce bias in estimates by accounting for covariates that could potentially distort the effect estimate. This statistical technique is worthwhile when the sample size is not too large, since it allows including a number of covariates in the model without broadening the confidence intervals of the estimates. This method matches subjects by a score that results from summing the values of all covariates we want to control for. [20]

To our knowledge, although other studies have examined the association between sleep and cardiometabolic risk in the same age-range, [21] no study has separately assessed the relationship between sleep patterns and MetS components in children. Moreover, the use of propensity score matching techniques supposes a greater control of covariates because they match similar individuals in terms of covariates.

This study was aimed at examining the influence of sleep characteristics (bedtime and amount of time in bed) in the cardiometabolic risk in 8-to-11-year-old Spanish children using propensity score matching techniques, and also to separately analyze the influence of these sleep parameters on each of the MetS components.

## Materials and methods

### Study design and population

A cross-sectional analysis including 210 children (55%, 116 girls) randomly selected among the participants in the baseline measurements from a cluster-randomized trial (collected September—November 2010) aimed to assess the effectiveness of a physical activity program on the prevention of excess weight in schoolchildren (MOVI-2) (Clinicaltrials.gov number NCT01277224). [22] The whole sample included 1,158 children aged 8–11 years from 20 public primary schools in the Province of Cuenca, Spain. The current analysis was conducted in a subsample of 210 children randomly selected (once it was known how many children finally participated in the study) in whom physical activity and sleep behaviour were measured by accelerometry. This subsample size was calculated considering a difference between control group and intervention group of a 10% in the proportion of children meeting physical activity recommendations (0.05 significance level and 0.8 of statistical power). After the analyses of accelerometer recordings, 64 subjects were excluded because they did not meet inclusion criteria. Included participants did not differ from those excluded in age, sex and lipid profile. The Clinical Research Ethics Committee of the Virgen de la Luz Hospital in Cuenca approved the study protocol. After obtaining the approval of the Director and Board of Governors of each school, a letter was sent to all parents of children in the fourth or fifth grade inviting them to a meeting, during which the study objectives were outlined and written approval for their children’s participation was requested. Informative talks, in which the schoolchildren were asked to collaborate, were then held in each class.

### Measurement

#### Anthropometrics

Trained nurses measured anthropometric variables and blood pressure. Data collection was performed at the schools during September 2010. Weight and height were measured twice at a 5-min interval. Weight was measured to the nearest 100 g using a calibrated digital scale (SECA model 861; Vogel & Halke, Hamburg, Germany), with children dressed lightly and without shoes. Height was measured to the nearest millimetre with a wall-mounted stadiometer, with children standing straight against the wall without shoes, to align the spine with the stadiometer. The head was positioned with the chin parallel to the floor. The mean of the two measurements of weight and height was used to calculate the body mass index (BMI) as weight in kilograms divided by the square of the height in meters (kg/m^2^). Waist circumference (WC) was determined by the average of two measurements taken with a flexible tape at the waist (at the midpoint between the last rib and the iliac crest). Fat mass percentage was estimated using a BC-418 bioimpedance analysis system (Tanita Corp., Tokyo, Japan). [23] Two readings were taken in the morning, after 12 hours fasting, under controlled temperature and humidity conditions, with the child without shoes, and after urination and a 15-min rest.

#### Resting blood pressure

Diastolic and systolic blood pressure (DBP and SBP) were determined by the average of two measurements taken at an interval of 5 min, with the subject resting for at least 5 min before the first measurement. The participant was seated in a quiet and calm environment, with the right arm placed in a semiflexed position at heart level. Blood pressure was measured by an automated procedure using the Omron M5-I monitor (Omrom Healthcare Europe BV, Hoofddorp, the Netherlands). The mean arterial pressure (MAP) was then calculated using the following formula: DBP + [0.333 x (SBP–DBP)].

#### Biochemical assessments

Blood samples were taken by puncturing the cubital vein, under standardized conditions, between 8:15 and 9:00 A.M., with the participant having fasted at least 12 hours beforehand. When the transfer of the samples to the laboratory took longer than 75 min, they were centrifuged in situ and transferred refrigerated.

The following variables were determined according to biochemical parameters: triglycerides (TGs) (glycerol phosphate oxidase-peroxidase [GPO-PAP] enzymatic method) and c-direct plus high-density lipoprotein (HDL). Lipid profile determinations were made on a weekend, in a MODULAR DPP from Roche Diagnostics, and insulin levels were assessed using an Immulite 2000 double system platform of Siemens.

#### Cardiometabolic risk index (CMRI)

This model includes a single variable for each of the four core components usually accepted in MetS: waist circumference (WC) for abdominal obesity, fasting insulin for insulin resistance, triglyceride/HDL cholesterol ratio (Trigly/HDL-C) for dyslipemia, and MAP for hypertension. [24]

We calculated a cardiometabolic risk index (CMRI) as the sum of the age- and sex-standardized scores of WC, fasting insulin, TG-to-HDL cholesterol ratio (TG/HDL-c), and MAP The validity of this index has been previously tested using confirmatory factor analysis. [24]

#### Evaluation of sleep patterns

Nocturnal sleep (duration and quality) was objectively assessed using accelerometry. Children were asked to wear an ActiGraph accelerometer monitor (GT3X+, Pensacola, FL, USA) tightly on the mid-axillary line of the right hip using an elastic belt for seven consecutive days (for the entire 24-hour period); they were only allowed to remove it during water activities (i.e. showering or swimming) or high contact activities (e.g. martial arts). When worn during sleep, the ActiGraph monitor can estimate sleep onset, sleep latency, wake after sleep onset (WASO), number and length of awakenings, sleep duration and sleep efficiency. Sleep latency is the length of time taken to fall asleep, calculated as the time between ‘lights off’ to the first period of 3 min of consecutive epochs scored as sleep. WASO is the number of minutes awake between sleep onset and time of final waking. Sleep efficiency is defined as the proportion of the estimated sleep periods spent asleep. Actigraphy data were collected in 60-second epochs and with a 30- Hertz sampling rate.

Parents and children were instructed to keep logs for bedtime (‘lights off’ and trying to sleep) and wake-up time (‘lights on’) during the week in which the monitor was worn. These self-reported bedtimes and wake-up times were used to estimate accelerometer determined sleep duration, allowing a visually inspected window up to 30 minutes with accelerometer registered data to avoid large inaccuracies in parent-reported times. There were no missing sleep log data for children included in this study. Parents were asked to record their children’s sleep logs each morning upon waking and to ensure the sleep logs were complete. Research staff checked children’s sleep logs when caregivers returned with the ActiSleep monitors. Information from sleep logs was not used to substitute missing actigraphy data.

At the end of the observation period, data were analysed using the ActiLife 6 software (the ActiGraph 2012, ActiLife version 6); for that, we used the ‘Sadeh’ sleep algorithm validated for children. [25]

The weekly average of sleep parameters was calculated in the proportion of five to two between weekdays (Sunday to Thursday) and weekend days (Friday and Saturday). Sleep parameters were only considered valid if they were measured for a minimum of three weekdays and one weekend day. More than eighty-five percent of individuals had valid sleep registrations for seven days and nights. The averages of sleep parameters data were calculated for children who meet the required days.

#### Cardiorespiratory fitness

Cardiorespiratory fitness was evaluated using the 20-min shuttle run test, which has been validated as a measure of maximal aerobic capacity in children. It was performed using the Léger protocol. [26] All tests were conducted by a trained research team that provided standardized encouragement for participants during all test phases. The equation of Leger et al. (1988) was used to estimate VO2max. Scores of the last stage number were converted to predict maximal oxygen uptake: VO2max (ml/kg/min) = 31.025 + 3.238 x (speed-km/h)– 3.248 x (age) + 0.1536 x (speed x age).

### Statistical analysis

The distribution of continuous variables was checked for normality before analyses; fasting insulin and TG/HDL-c were normalized by a natural logarithm transformation.

Partial correlation coefficients controlling for age were estimated to examine the relationship between sleep parameters and cardiometabolic risk factors.

Children were categorized as normal weight, overweight, or obese according to sex- and age-specific cut-offs defined by Cole and Lobstein. [27] Sleep parameters were categorized in quartiles.

ANCOVA models were fitted to test differences in MetS (CMRI and individual cardiometabolic risk factors) across categories of total time in bed (TTB) and bedtime (< percentile 25, percentile 25–75 and > percentile 75) controlling for age (model 1), also including wake-up time in a second step (model 2). Pairwise post-hoc hypotheses were tested using the Bonferroni test. These statistical analyses were performed using the IBM SPSS 22 software (SPSS, Inc., Chicago, IL).

Histograms depicting TTB and the different cardiometabolic risk parameters were used to approximately determine the cut off in which cardiometabolic risk substantially increased. After that, a propensity score derivation model was built with TTB as the dependent variable, the cut off (9 h 15 min) categorized the sample in two groups (time in bed >9.15 and time in bed <9.15), and four MetS-related covariates (age, gender, weight status, sleep efficiency) and cardiorespiratory fitness as matching covariates. These covariates and the MetS index in the matched group were compared between time in bed groups using the Student’s t-test for continuous variables and the chi-square test for categorical variables. [28] These statistical analyses were performed with StataSE software, version 14 (StataCorp).

A criterion for statistical significance of p ≤ 0.05 was used.

## Results

Characteristics of the study sample are presented as mean and standard deviation (SD) in Table 1. We restricted our analyses to participants with data available for all variables studied. Of 1,158 participants in MOVI-2, 146 children (54.8% girls) met all inclusion criteria. Means of fat mass percentage, sleep efficiency and total time in bed were greater in girls than in boys; conversely, MAP values were higher in boys (p <0.05).

**Table 1:** Characteristics of the study sample.

	Total (n = 146)	Boys (n = 66)	Girls (n = 80)	p
Age (years)	9.40 (0.74)	9.35 (0.79)	9.44 (0.69)	0.470
Weight (kg)	36.38 (9.01)	37.19 (10.16)	35.71 (7.95)	0.326
Height (cm)	138.92 (6.85)	138.18 (6.79)	139.53 (6.89)	0.238
BMI (kg/m^2^)	18.71 (3.81)	19.31 (4.43)	18.22 (3.17)	0.085
Fat mass (%)	25.10 (6.80)	23.86 (7.91)	26.12 (5.57)	**0.045**
VO2max	43.16 (2.99)	42.77 (2.83)	43.50 (3.11)	0.165
WC (cm)	67.78 (9.80)	68.88 (11.22)	66.87 (8.42)	0.219
Fasting insulin (mg/dL)	0.85 (0.23)	0.83 (0.23)	0.87 (0.23)	0.278
TG/HDL-c (mg/dL)	0.02 (0.26)	-0.01 (0.28)	0.04 (0.24)	0.301
MAP (mmHg)	75.48 (6.82)	76.78 (6.39)	74.40 (7.01)	**0.036**
CMRI	0.09 (1.76)	0.24 (1.84)	-0.04 (1.69)	0.340
In Bed (hh:mm)	22:53 (00:36)	22:57 (00:40)	22:50 (00:32)	0.241
Out Bed (hh:mm)	08:27 (00:26)	08:23 (00:28)	08:29 (00:24)	0.184
Latency (min)	9.61 (6.60)	10.13 (7.71)	9.18 (5.54)	0.390
Efficiency (%)	92.84 (3.12)	92.24 (3.46)	93.34 (2.74)	**0.034**
TTB (min)	573.59 (36.70)	566.64 (40.05)	579.33 (32.84)	**0.037**

Data are presented by mean (SD). Boldface type indicates statistical significance p < 0.05.

Abbreviations: BMI, body mass index; WC, waist circumference; TG/HDL-c, logarithm of triglycerides/high density lipoprotein cholesterol; MAP, mean arterial pressure; CMRI, cardiometabolic risk index; TTB, total time in bed.

Mean differences in WC triglyceride/HDL-c ratio, insulin, and MAP by TTB categories, controlling for age, are shown in Table 2. Overall, for the whole sample, children in the lower TTB category showed worse values in all cardiometabolic risk factors than their peers in other TTB categories, except for fasting insulin, in which no differences were found. Likewise, for boys, the cardiometabolic profile was significantly worse in the lower TTB category, although in MAP, differences were not statistically significant. In girls, no significant differences were found in any of the cardiometabolic risk parameters. Regarding the CMRI, children in the lowest TTB category scored worse than those in other TTB categories, although in girls, the difference did not achieve statistical significance.

**Table 2:** Mean differences in cardiometabolic risk factors by total time in bed categories controlling for age.

Total Time in Bed					
	< P25	P 25–75	>P75	p	Bonferroni Post-hoc
**Total simple** (n = 146)	n = 36	n = 73	n = 37		
WC (cm)	72.22 (11.47)	66.03 (8.48)	66.91 (9.40)	**0.014**	1>2
Log TG/HDL-c (mg/dL)	0.15 (0.29)	-0.02 (0.23)^b^	-0.03 (0.26)	**0.006**	1>2;1>3
Log insulin (mg/dL)	0.93 (0.21)	0.81 (0.26)^b^	0.86 (0.17)	0.064	
MAP (mmHg)	78.18 (5.62)	73.98 (6.45)	75.80 (7.83)	**0.023**	(1>2)
CMRI	1.00 (1.83)	-0.29 (1.64)^b^	-0.06 (1.65)	**0.002**	1>2;1>3
**Boys** (n = 66)	n = 22	n = 29	n = 15		
WC (cm)	74.76 (12.63)	65.24 (8.39)	67.28 (10.94)	**0.036**	1>2
Log TG/HDL-c (mg/dL)	0.18 (0.33)	-0.08 (0.21)	-0.13 (0.17)	**0.001**	1>2;1>3
Log insulin (mg/dL)	0.94 (0.23)	0.74 (0.24)	0.83 (0.15)	**0.018**	1>2
MAP (mmHg)	79.08 (5.93)	75.54 (5.47)	75.80 (8.03)	0.286	
CMRI	1.31 (2.06)	-0.33 (1.46)	-0.22 (1.57)	**0.004**	1>2;1>3
**Girls** (n = 80)	n = 14	n = 44	n = 22		
WC (cm)	68.23 (8.28)	66.55 (8.60)	66.65 (8.46)	0.800	
Log TG/HDL-c (mg/dL)	0.09 (0.21)	0.02 (0.23)^c^	0.04 (0.30)	0.661	
Log insulin (mg/dL)	0.91 (0.17)	0.85 (0.26)^c^	0.88 (0.17)	0.716	
MAP (mmHg)	76.76 (4.98)	72.95 (6.89)	75.80 (7.88)	0.132	
CMRI	0.52 (1.33)	-0.27 (1.77)^c^	0.05 (1.72)	0.321	

Data are presented by mean (SD).

Abbreviations: WC, waist circumference; log TG/HDL-c, logarithm of triglycerides/high density lipoprotein cholesterol; log insulin, logarithm of fasting insulin; MAP, Mean Arterial Pressure; CMRI, Cardiometabolic Risk Index.

Categories of Total Time in Bed represent the 1st, 2nd and 3rd (2), and 4th quartiles. Bonferroni post-hoc test was used in all the pairwise mean comparisons statistically significant as shown in boldface type (p<0.05).

^b^ n = 72.

^c^ n = 43.

A propensity score derivation model was estimated matching the 32 subjects with TTB >9 h 15 min with 32 subjects with TTB <9 h 15 min. Table 3 shows the mean differences (percentage when appropriate) in measured covariates for TTB groups after propensity score matching. In the CMRI mean, differences were observed when TTB was below 9h 15 min (p ≤ 0.05). Additionally, when the propensity score was applied separately by sex, differences in insulin and ratio triglyceride/HDL cholesterol were found between TTB groups in boys, and no differences were found in girls.

**Table 3:** Differences of metabolic syndrome index and some of measured covariates between time in bed groups before and after matching.

	Before matching			After matching		
	Time in bed			Time in bed		
Subject-related variables	<9h15min	>9h15min		<9h15min	>9h15min	
**TOTAL**	n = 36	n = 109	p	n = 32	n = 32	p
**Matching variables**						
Age	9.55 ±0.73	9.34 ±0.73	0.139	9.47 ±0.73	9.72 ±0.71	0.116
Boys	22(61.11)	44 (40.00)	**0.027**	24 (75.00)	20 (62.50)	0.288
Overweight	19 (52.78)	33 (30.00)	**0.013**	18 (59.37)	17 (53.12)	0.324
Efficiency <91%	5 (13.89)	31 (28.18)	0.068	8 (25.00)	4 (12.50)	0.371
VO2max	43.17 ±2.60	43.15 ±3.12	0.975	43.17 ±2.60	43.50 ±2.05	0.607
**Metabolic Syndrome variables**						
MAP	78.18 ±5.62	74.59 ±6.96	**0.006**	77.84 ±5.54	76.08 ±6.11	0.233
Waist circumference	72.22 ±11.47	66.32 ±8.77	**0.001**	71.94 ±11.33	68.61 ±8.44	0.188
Fasting Insulin	9.36 ±4.07	7.67 ±4.09	**0.033**	9.16 ±4.02	7.30 ±4.91	0.105
TG/HDL-c	1.63 ±1.16	1.07 ± 0.86	**0.002**	1.51 ±0.93	1.14 ±0.74	0.085
CMRI	1.00 ± 1.83	-0.21 ± 1.64	**<0.001**	0.95±1.83	- 0.06±4.65	**0.020**
**BOYS**	n = 22	n = 44	p	n = 20	n = 20	p
**Matching variables**						
Age	9.64 ±0.79	9.20 ±0.76	**0.036**	9.50 ±0.69	9.60 ±0.61	0.809
Overweight	13 (59.09)	14 (31.82)	**0.034**	11 (55.00)	11 (55.00)	1.000
Efficiency <91%	5 (22.73)	16 (36.36)	0.262	4 (20.00)	4 (20.00)	1.000
VO2max	42.77 ±2.43	42.77 ±3.03	0.995	42.77 ±2.43	43.34 ±2.13	0.607
**Metabolic Syndrome variables**						
MAP	79.08 ±5.92	75.63 ±6.37	**0.037**	78.55 ±5.83	79.27 ±6.44	0.714
Waist circumference	74.76 ±12.63	65.94±9.26	**0.002**	73.57 ±12.64	70.75 ±10.80	0.452
Fasting Insulin	9.85 ±4.73	6.62 ±3.10	**0.001**	9.55 ±4.63	6.13 ±3.20	**0.010**
TG/HDL-c	1.86 ±1.35	0.83 ± 0.38	**<0.001**	1.62 ±1.05	0.98 ±0.40	**0.016**
CMRI	1.31 ± 2.06	-0.29 ± 1.48	**<0.001**	1.15±2.08	0.06±1.48	0.065
**GIRLS**	n = 14	n = 65	p	n = 12	n = 12	P
**Matching variables**						
Age	9.42 ±0.65	9.44 ±0.70	0.958	9.42 ±0.67	9.67 ±0.49	0.308
Overweight	6 (42.86)	19 (29.23)	0.320	6 (50.00)	6 (50.00)	1.000
Efficiency <91.%	0 (0.00)	15 (23.07)	**0.045**	0 (0.00)	0 (0.00)	1.000
VO2max	43.83 ±2.85	43.43 ±3.17	0.681	43.84 ±2.85	44.10 ±3.77	0.850
**Metabolic Syndrome variables**						
MAP	76.76 ±4.98	73.90 ±7.30	0.167	76.65 ±5.03	75.51 ±9.18	0.712
Waist circumference	68.23 ±8.28	66.58±8.49	0.508	69.21 ±8.54	65.69 ±5.62	0.452
Fasting Insulin	8.59 ±2.73	8.38 ±4.53	0.866	8.52 ±2.81	7.48 ±3.24	0.414
TG/HDL-c	1.28 ±0.67	1.23 ± 1.04	0.861	1.33 ±0.69	0.92 ±0.29	0.073
CMRI	0.52 ± 1.33	-1.16 ± 1.75	0.176	0.63 ±1.35	0.50±1.53	0.835

Data are reported as mean ± SD or number (proportion). Efficiency <91% (Percentage corresponding to the 1st quartile).

Abbreviations: TG/HDL-c, logarithm of triglycerides/high density lipoprotein cholesterol; MAP, Mean Arterial Pressure; CMRI, Cardiometabolic Risk Index.

Bedtime was categorized by quartiles too, and we defined early bedtime as the lowest quartile (score ≤ 22:30 h), normal bedtime as the 2^nd^ to 3^rd^ quartile (score 22:30 h to 23:15 h), and late bedtime as the highest quartile (score ≥ 23:15 h). When we analysed the mean differences in cardiometabolic risk factors and CMRI by bedtime categories (Table 4), we observed worse values in cardiometabolic profile and CMRI in children who went to bed later, but these differences were statistically significant only for the TG/HDL-c ratio, fasting insulin, and the CMRI (p < 0.05) when we adjusted for age (Model 1); however, when we also included age and wake-up time in the model (Model 2), differences disappeared for fasting insulin.

**Table 4:** Mean differences in cardiometabolic risk factors by bedtime categories.

	Bedtime				
	< P25	P25-P75	>P75	p value Model 1	p value Model 2
n	36	74	36		
Evening time	<22:30h	22:30–23:15h	>23:15h		
**WC (cm)**	65.10 (8.52)	67.95 (9.49)	70.10 (11.16)	0.141	0.066
**Log TG/HDL-c (mg/dL)**	-0.08 (0.18)	0.04 (0.29)	0.08 (0.24)	**0.042**	**0.026** (1<3)
**Log Insulin (mg/dL)**	0.77 (0.17)	0.86 (0.25)	0.91 (0.23)	**0.044** (1<3)	0.065
**MAP (mmHg)**	76.06 (7.01)	74.36 (7.00)	77.20 (5.91)	0.111	0.110
**CMRI**	-0.53 (1.45)	0.12 (1.85)	0.65 (1.72)	**0.023** (1<3)	**0.013** (1<3)

Data are presented by mean (SD).

Abbreviations: P, percentile; WC, waist circumference; log TG/HDL-c, logarithm of triglycerides/high density lipoprotein cholesterol; log insulin, logarithm of fasting insulin; MAP, mean arterial pressure, CMRI, cardiometabolic risk index.

Bonferroni post-hoc test was used in all the pairwise mean comparisons statistically significant as shown in boldface type (p < 0.05). Model 1, adjusted by age; Model 2, adjusted by age and wake time.

## Discussion

A recent meta-analysis has provided evidence regarding the association between sleep characteristics and MetS in children. [29] However, the current study is, to our knowledge, the first to analyse in the same study, on the one hand, the relationship between cardiometabolic risk profile and categories of TTB using a propensity score analysis and, on the other hand, the influence of bedtime on MetS controlling for wake-up time. Moreover, in contrast with most studies using the homeostasis model (HOMA-IR) as an indicator of insulin resistance, we used a fasting insulin assessment because it can be a more sensitive indicator of insulin resistance even in children without elevated glycaemia. [24]

In both children and adults, a relationship between an inadequate sleep pattern and cardiometabolic risk has been proposed. [30] Regarding abdominal obesity, weak evidence from observational studies has associated short sleep time with increased caloric intake [31, 32] and weight gain. Potential explanations for this increased food intake include more time for food consumption, alterations in appetite regulation hormones and changes in food preferences in favour of energy dense foods. Also, a reduction in physical activity has been proposed as a potential mechanism linking sleep reduction with abdominal obesity. However, evidence is inconclusive and different results for boys and girls have been reported. Our data support that those children that sleep less than 9h 15 min have a significantly greater waist circumference, although statistical significance disappeared after covariates matching by using propensity score analysis, probably because of the small number of matched pairs.

Observational studies have associated shorter sleep duration with increased insulin resistance indicators. However, in most studies, this association disappeared after controlling for adiposity measures. Overall, studies supporting this association are scarce and current evidence suggests that short sleep time as well as sleep problems are associated with hyperinsulinemia in children; however, a U shaped association could be behind all of these controversial findings. [33] As in the whole cardiometabolic risk profile, our data support that sleep restriction is associated with increased insulin resistance, although statistical significance disappeared when the sample size was limited for propensity score matching.

The available literature shows inconsistencies regarding associations between sleep duration and blood lipids in children. Although some studies have suggested that sleep problems and, in general, bad sleep quality are associated with adverse lipid profile, in most studies, this association disappeared after controlling for an adiposity indicator. In addition, sex differences have been reported with regards to the influence of sleep quality on children’s lipid profile. [30] Our results showed that shorter TTB was associated with a higher triglycerides/HDL-c ratio in boys but not in girls, being significant only in boys. Moreover, we found a significant positive association with bedtime, which was maintained when adjusting for wake-up time. The mechanisms that have been suggested to explain the association between inadequate sleep and dyslipidemia include increased food intake, physical activity restrictions because of daytime fatigue, changes in glucose homeostasis and adiponectin levels. [34]

In general, studies have reported inconsistent results in the relationship between sleep patterns and blood pressure (BP); however, some studies have showed a modest beneficial effect of longer sleep duration on BP. [30] Our results showed a significant increase in BP in children with a short sleep time; however, as has been noted above, these differences disappeared when subjects were matched using propensity scores, probably because of sample size issues. Apart from physical activity restrictions and unhealthy diet, other proposed pathways linking shorter sleep time with elevated BP such as circadian mismatching, changes in sympathetic nervous system (SNS) activity, and hyperinsulinemia-related sodium retention. [35, 36]

Although the influence of irregular bedtimes on children’s academic achievement and behavioural problems has been repeatedly reported, evidence regarding the independent influence of bedtime in cardiometabolic risk is lacking. Our data suggest that an earlier bedtime (before 22:30 h) is associated with a lower global cardiometabolic risk and waist circumference even after controlling for wake-up time. Since this dimension of sleep is probably that in which prevention endeavours are more efficient, it seems thoughtful to implement parenting educational interventions that address sleep hygiene as a pivotal health behaviour. [37]

Nonetheless, these results should be interpreted with caution given the limitations of this study. First, causal relationship between an inadequate sleep pattern and cardiometabolic risk cannot be estimated due to the inherent temporal ambiguity of cross-sectional studies. Second, accelerometers are designed for measuring acceleration and detecting movement, therefore an accurate assessment of sleep and waking state may not be appropriate with these devices. In addition, it has been observed that waist-worn accelerometers overestimate sleep efficiency compared to wrist-worn accelerometers. [38]

Even considering the lack of statistical significance because the small sample size limits the robustness of our conclusions, our data suggest that children’s sleep hygiene should be incorporated in parenting educational programs.

## Conclusions

Our study shows an association between short sleep duration and cardiometabolic risk, in such a way that children who sleep less than 9 hours 15 minutes have a worse cardiometabolic profile. In addition, we have observed that bedtime may independently influence cardiometabolic risk. These findings should be confirmed with longitudinal research that consistently verifies the association between sleep duration and cardiometabolic risk.
